# Sexual dysfunction following surgery for rectal cancer - a clinical and neurophysiological study

**DOI:** 10.1186/1756-9966-28-128

**Published:** 2009-09-17

**Authors:** Alberto Pietrangeli, Patrizia Pugliese, Maria Perrone, Isabella Sperduti, Maurizio Cosimelli, Bruno Jandolo

**Affiliations:** 1 Service of Neurology, Regina Elena Cancer Institute, Rome, Italy; 2 Service of Psychology, Regina Elena Cancer Institute, Rome, Italy; 3 Service of Biostatistics, Regina Elena Cancer Institute, Rome, Italy; 4 Department of Surgery, Regina Elena Cancer Institute, Rome, Italy

## Abstract

**Background:**

Sexual dysfunction following surgery for rectal cancer may be frequent and often severe. The aim of the present study is to evaluate the occurrence of this complication from both a clinical point of view and by means of neurophysiological tests.

**Methods:**

We studied a group of 57 patients submitted to rectal resection for adenocarcinoma. All the patients underwent neurological, psychological and the following neurophysiological tests: sacral reflex (SR), pudendal somatosensory evoked potentials (PEPs), motor evoked potential (MEPs) and sympathetic skin responses (SSRs). The results were compared with a control group of 67 rectal cancer patients studied before surgery. Only 10 of these patients could be studied both pre- and postoperatively. 10 patients submitted to high dose preoperative chemoradiation were studied to evaluate the effect of this treatment on sexual function. Statistical analysis was performed by means of the two-tailed Student's *t *test for paired observations and *k *concordance test.

**Results:**

59.6% of patients operated reported sexual dysfunction, while this symptom occurred in 16.4% in the control group. Moreover, a significantly higher rate of alterations of the neurophysiological tests and longer mean latencies of the SR, PEPs, MEPs and SSRs were observed in the patients who had undergone resection. In the 10 patients studied both pre and post-surgery impotence occurred in 6 of them and the mean latencies of SSRs were longer after operation. In the 10 patients studied pre and post chemoradiation impotence occurred in 1 patient only, showing the mild effect of these treatments on sexual function.

**Conclusion:**

Patients operated showed severe sexual dysfunctions. The neurophysiological test may be a useful tool to investigate this complication. The neurological damage could be monitored to decide the rehabilitation strategy.

## Background

Sexual dysfunction following surgery for rectal cancer is variable and the literature of the past reported rate until 100% of the patients. [1-9]. In the last report [9] the rate of total impotence in men is 32%. The explanation is a damage of the pelvic autonomic nerves with consequence on sexual functioning in males and females (erection, ejaculation, drive).

Neurophysiological techniques such as electromyography of the pelvic floor, examination of the sacral reflex (SR), pudendal somatosensory evoked potentials (PEPs), motor evoked potentials (MEPs) and sympathetic skin responses (SSRs), have been employed in recent years to evaluate this complication [10-12].

The aim of the present study was therefore to evaluate the occurrence of sexual dysfunction from both a clinical point of view and by means of neurophysiological tests in patients submitted to surgery for rectal cancer.

## Methods

We studied a group of 57 patients (43 males and 14 females, mean age 57.9 years, range 29-72 years) with rectal cancer who, over the past three years, underwent low anterior resection with total mesorectal excision and with sympathetic and parasympathetic nerve-sparing technique. Tumor location was defined by the distance from the anal verge. The mean distance was cm. 6.53 (range cm. 2-10).

10 patients were treated with preoperative chemoradiation. No surgical complication and relapse were diagnosed.

All the examinations were carried out with informed consent and approved by the ethical commission.

A detailed history of the patients' sexual functions both pre- and postoperatively was obtained using the International Index of Erectile Function [13]. The sexual functioning was also evaluated with a structured interview in agreement to the criteria of DSM-IV (American Psychiatric Association) and with neurophysiological tests. The frequency of copulation, ejaculation and penile erection was documented in males, while sexual desire, excitement, drive and orgasm were recorded in the females. All the patients were submitted to general physical and neurological examinations. No patient showed signs or symptoms related to other neurological disorders. The patients underwent psychological tests (psychodynamic interview, Hospital Anxiety and Depression Scale of Zigmond and Snaith) [14]. Those with psychogenic impotence, sexual psychological dysfunctions and other psychiatric symptoms were excluded from the study.

The neurophysiological examination was conducted according to the following procedures established in the literature. Normal values were fixed comparing literature data with values from normal subjects of our series.

1) SR: recordings with coaxial electrode needle inserted in the anal sphincter; stimulation with bipolar electrode on the penis or clitoris (proximal cathode), intensity three times the sensory threshold. The shortest latency of the first response (R1) on eight stimulations was chosen.

2) PEPs: recordings with monopolar needle electrodes in Cz' (2 cm behind Cz) with frontal reference Fpz; stimulation with bipolar electrodes on the penis or clitoris, intensity twice the sensory threshold; averaging 250 stimuli, frequency 3 Hz, filter bandpass of 20-200 Hz.

3) MEPs: recordings with coaxial needle electrodes (filters 20-10,000 Hz) from the anal sphincter in contraction; magnetic cortical stimulation at vertex was carried out with a Novametrix Magstim 200 (coil diameter: 9 cm; maximum peak value of magnetic field: 2 tesla) at 95% power level.

4) SSRs: recordings with Ag/AgCl disk electrodes filled with conductive jelly placed on perineum (active) and pubis, stimulation on the right median nerve at the wrist with bipolar electrode (distal cathode), intensity twice the sensory threshold: the shortest latency of the first response on eight stimulations delivered at random every 20 sec was chosen. Recordings could be evaluated in only 17 patients.

Not all the patients completed these four tests because of technical difficulties following the local state of the skin unable to support electrodes. Data are showed in tables 1 and 2.

**Table 1:** Results for the overall control group (n. 67)

	**age**	**SR**	**PEP**	**MEP**	**SSR**	**sexual function**
Normal values		<38 msec	<45 msec	<31 msec	<1.7 sec	
Mean values	56.9	33.47(sd 6.2)	41.59(sd 9.54)	27.44(sd 4.83)	1.66 (sd 0.21)	
**Abnormal (%)**		**12 (17.9%)**	**11 (18.9%)**	**12 (23.5%)**	**20 (45.5%)**	**11 (16.4%)**
Normal		55 (82.1%)	47 (81%)	39 (76.5%)	24 (54.5%)	56 (83.6%)
Not evaluated		0	9	16	23	0

**Table 2:** Results for the overall postoperative group(n. 57)

	**age**	**SR**	**PEP**	**MEP**	**SSR**	**sexual function**
Normal values		<38 msec	<45 msec	<31 msec	<1.7 sec	
Mean values	57.9	36.57(sd 9.54)*	41.92(sd 3.95)	28.08(sd 3.12)	1.81(sd 0.22)***	
**Abnormal**		18 ** (33.3%)	**10 (21.7%)**	**13 (33.3%)**	20 **** (71.4%)	34***** (59.6%)
Normal		36 (66.7%)	36 (78.3%)	26 (66.7%)	8 (28.6%)	23 (40.4%)
Not evaluated		3	11	18	29	0

* p ≤ 0.04 *** p ≤ 0.009 ***** p ≤ 0.0001

** p ≤ 0.05 **** p ≤ 0.03

**K concordance test**:

SR vs sexual dysfunction k = 33 p ≤ 0.006

SSR vs sexual dysfunction k = 38 p ≤ 0.02

SR = sacral reflex PEP = pudendal somatosensory evoked potentials MEP = motor evoked potentials SSR = sympathetic skin responses

The results were compared with a second group of 67 patients (43 males and 24 females, mean age 56.9 years, range 19-73 years) to be submitted to surgery for rectal cancer. This group of patients was similar to the first one for age, sex and highness. Only 10 of these patients could be studied both pre- and postoperatively (table 3 and 4). 10 patients submitted to high dose preoperative chemoradiation were studied to evaluate the effect of this treatment on sexual function (table 5 and 6).

**Table 3:** Results for the preoperative group (n. 10)

	**Age**	**SR**	**PEP**	**MEP**	**SSR**	**Sexual function**
Normal values		<38 msec	<45 msec	<31 msec	<1.7 sec	
Mean values	56	34.08(sd 5.18)	40.35(sd 3.84)	28.40(sd 3.07)	1.75(sd 0.17)	
**Abnormal**		**2 (20%)**	**1 (12.5%)**	**1 (20%)**	**2 (28.6%)**	**2 (20%)**
Normal		8 (80%)	7 (87.5%)	4 (80%)	5 (71.4%)	8 (80%)
Not evaluated		0	2	5	3	0

**Table 4:** Results for the postoperative group (n. 10)

	**age**	**SR**	**PEP**	**MEP**	**SSR**	**Sexual function**
Normal values		<38 msec	<45 msec	<31 msec	<1.7 sec	
Mean values	58	35.63(sd 8.10)	42.35(sd 3.54)	25.78(sd 2.72)	2.33(sd 0.49)*	
**Abnormal**		**2 (20%)**	**3 (37.5%)**	**0**	6 ** (85.7%)	6 *** (60%)
Normal		8 (80%)	5 (62.5%)	5 (100%)	1 (14.3%)	4 (40%)
Not evaluated		0	2	5	3	0

* p ≤ 0.04 ** p ≤ 0.12 *** p ≤ 0.12

SR = sacral reflex PEP = pudendal somatosensory evoked potentials MEP = motor evoked potentials SSR = sympathetic skin responses

**Table 5:** Results for the prechemoradiation group (10 patients)

	**Age**	**SR**	**PEP**	**MEP**	**SSR**	**Sexual function**
Normal values		<38 msec	<45 msec	<31 msec	<1.7 sec	
Mean values	57.5	34.76(sd 4.33)	43(sd 3.51)	24.64(sd 4.64)	1.69(sd 0.09)	
**Abnormal**		**2 (20%)**	**2 (28.6%)**	**1 (14.3%)**	**1 (25%)**	**0 (0%)**
Normal		8 (80%)	5 (71.4%)	6 (85.7%)	3 (75%)	10 (100%)
Not evaluated		0	3	3	6	0

**Table 6:** Results for the postchemoradiation group (10 patients)

	**Age**	**SR**	**PEP**	**MEP**	**SSR**	**Sexual function**
Normal values		<38 msec	<45 msec	<31 msec	<1.7 sec	
Mean values	57.8	33.58(sd 5.82)	42.43(sd 3.27)	27.6(sd 3.05)	1.94(sd 0.18)*	
**Abnormal**		**3 (30%)**	**2 (28.6%)**	**1 (14.3%)**	**3 (75%)**	**1 (10%)**
Normal		7 (70%)	5 (71.4%)	6 (85.7)	1 (25%)	9 (90%)
Not evaluated		0	3	3	6	0

* p ≤ 0.04

SR = sacral reflex PEP = pudendal somatosensory evoked potentials MEP = motor evoked potentials SSR = sympathetic skin responses

Statistical analysis was performed by means of the two-tailed Student's *t *test for paired observations and *k *concordance test.

## Results

Overall 59.6% of the patients submitted to resection had sexual impotence. In the control group this complication occurred in only 16.4% (p ≤ 0.0001) (tables 1 - 2). Abnormal values were observed in 33.3% of the patients submitted to the SR test (p = 0.05), in 21.7% of the patients submitted to PEPs, in 33.3% of the patients submitted to MEPs and in 71.4% of the patients submitted to SSR (p ≤ 0.03), showing a higher incidence of alterations than in the control group. The mean latencies of the SR, PEPs, MEPs and SSRs were also longer (SSRs p ≤ 0.009) (tables 1 - 2).

In the 10 patients studied both pre and post-surgery impotence occurred in 6 of them and the mean latencies of SSRs were longer after operation (p ≤ 0.04) (tables 3 - 4). In the 10 patients studied pre and post chemoradiation impotence occurred in 1 patient only, showing the mild effect of these treatments on sexual function (tables 5 - 6)

## Discussion

Many authors consider neurophysiological testing unreliable to study sexual dysfunctions. In a series of patients with sexual and urogenital complaints Delodovici found abnormal PEPs in a very small proportion of patients (8%), according to the hypothesis of a predominant involvement of small fibers in these patients [15]. In a report of the Therapeutics and Technology Assessment Subcommittee of the American Academy of Neurology, the sensitivity and specificity of the PEPs in male sexual dysfunction is considered scarce. This test must therefore be correlated with other information to evaluate the impotent patient [16].

In a patient with partial resection of the presacral nerves and a radical cystectomy, Opsomer observed normal PEPs and alterations of the MEPs and the SR [17]. Rossini emphasizes the intersubject and intrasubject variability of SSRs, representing a severe limitation to the clinical applications of this test. This author suggests estimating the latency differences and amplitude ratio between the two body sides [18]. Ertekin emphasizes the usefulness of PEPs in spinal cord/cauda equina injuries and the superiority of the SR in diabetic impotence and in cauda/conus lesions.[19] In a study of 30 men with erectile impotence, Kunesck recommends the use of various tests for autonomic dysfunction [20], while Opsomer suggests employing a combination of cortical evoked potentials and sacral latency testing to accurately locate the lesion level.

In a recent study, we observed similar alterations in patients operated upon for colon and rectal cancer, but with a lower incidence of clinical and neurophysiological abnormalities, suggesting a minor frequency of sexual dysfunctions in colon cancer surgery [21].

In the present study the clinical value of neurophysiological tests to study sexual dysfunctions in patients undergoing surgery for rectal cancer is further confirmed with statistical significance for SSR, reflecting a local autonomic damage. The sacral reflex abnormalities found in post-operative group demonstrated the anatomical alterations of pelvic floor without specific involvement of small fibers. The lack of significant differences of PEPs and MEPs showed the integrity of ascending and descending pathways.

More significant data could be obtained from clinical and neurophysiogical examinations conducted according to a strict schedule: before surgery and at least every 6 months afterwards with the aim to evaluate the reversibility of the neuropathy. Unfortunately, an electrophysiological test battery is difficult to conduct in the follow-up of cancer patients and consequently the dropout rate is very high.

## Conclusion

This study confirms the helpful use of these tests in the study of sexual dysfunctions in rectal cancer surgery. This monitoring could be extended to all patients operated for cancer of the pelvic floor.

These tests could be a further aid in monitoring the post-surgery sexual dysfunction and its improvement to decide the best strategy in sexual rehabilitation.

The intraoperative recording of both the sacral reflex and anal MEP can be proposed in monitoring the integrity of pelvic floor somatic nerves during surgery but cannot be a specific test for sexual functions controlled by autonomic pathways.

Today sexual activity is considered a very important area of quality of life, therefore more efforts must be given to prevent this complication and to improve prognosis of patients.

## Competing interests

The authors declare that they have no competing interests.

## Authors' contributions

AP, PP, MP, MC, BJ participated in study in equal part.

IS carried out the statistical analysis.

All authors have read and approved the manuscript.
